# LigpKa – a database of pKa values for small molecule ligands designed for the use in structure-based pKa calculations

**DOI:** 10.1186/1758-2946-3-S1-P21

**Published:** 2011-04-19

**Authors:** P Czodrowski, CR Søndergaard, S Dohm, G Klebe, JE Nielsen

**Affiliations:** 1 Merck KGaA, Frankfurter Straße 250, D-64293 Darmstadt, Germany; 2 Philipps-University Marburg, Institute of Pharmaceutical Chemistry, Marbacher Weg 6, 35032 Marburg, Germany; 3 Department of Chemistry, University of Copenhagen, Universitetsparken 5, 2100 Copenhagen, Denmark; 4 School of Biomolecular and Biomedical Science, Conway Institute, UCD, Dublin 4, Ireland

## 

Electrostatic forces play a large role in determining the strength of protein-ligand interactions, and the calculation of pKa value shifts upon ligand binding is therefore an important component of any accurate protein-ligand binding calculation. However, such pKa calculations are rarely performed in virtual screening experiments due to the unavailability of ligand solution pKa values and the difficulty in generating the required charge distributions for each ligand protonation state. We present a freely available web-based database of small-molecule pKa values that automates the task of retrieving protonation states and solution pKa values for small-molecule ligands so that these can be used with tools such as PRODRG and PDB2PQR for generating the necessary parameters for a structure-based pKa calculation of a protein-ligand complex. The database contains a total of 348 pKa values and represents a significant step towards performing accurate automated pKa value calculations in virtual screening experiments.

